# Patellar tilt correction magnitude, rather than residual tilt, predicts functional outcome after isolated medial femoropatellar ligament reconstruction

**DOI:** 10.1002/jeo2.70874

**Published:** 2026-08-10

**Authors:** Ella George, Pierre‐Henri Vermorel, Frédéric Farizon, Rémi Philippot, Thomas Neri

**Affiliations:** ^1^ Department of Orthopaedic Surgery University Hospital Centre of Saint‐Étienne Saint‐Étienne France; ^2^ Inter‐University Laboratory of Human Movement Science University Lyon—University Jean‐Monnet Saint‐Étienne Saint‐Étienne France

**Keywords:** knee surgery, MPFL reconstruction, patellar tilt, patellofemoral instability, sports traumatology

## Abstract

**Purpose:**

Patellar tilt is considered a key factor in patellofemoral instability, with a commonly accepted pathological threshold of 20° on computed tomography (CT) scan assessment. However, the clinical relevance of correcting tilt below this threshold remains unclear. This study aimed to determine whether functional improvement after isolated medial patellofemoral ligament (MPFL) reconstruction is better associated with achieving a postoperative tilt <20° or with the magnitude of tilt correction.

**Methods:**

A retrospective study was conducted on 182 patients who underwent isolated MPFL reconstruction. Functional outcomes, including International Knee Documentation Committee (IKDC) and Kujala scores, were collected preoperatively and at final follow‐up. Patellar tilt was measured on CT scan preoperatively and at 6 months postoperatively, with both contracted and relaxed quadriceps. Patients were analysed according to postoperative tilt thresholds and correction differentials (diffQC and diffQR). Statistical analysis included group comparisons, correlation analyses, analysis of variance across correction categories and generalized linear models.

**Results:**

Patellar tilt on CT scan was significantly reduced postoperatively in both contracted and relaxed quadriceps across all groups (*p* < 0.001), with significant improvements in IKDC and Kujala scores at last follow‐up (*p* < 0.001). No significant differences in IKDC or Kujala scores were observed between patients with postoperative tilt <20° and >20° (all *p* > 0.05). No correlation was found between functional outcomes and isolated preoperative or postoperative tilt values. In contrast, greater correction magnitude was significantly associated with improved functional outcomes. A progressive increase in IKDC and Kujala scores was observed with increasing correction, with maximal gains for corrections ≥20°. This relationship was consistent for both contracted and relaxed quadriceps (all *p* < 0.001).

**Conclusion:**

Functional improvement after isolated MPFL reconstruction was more strongly associated with the magnitude of patellar tilt correction than with achieving a specific postoperative tilt threshold. Corrections exceeding 15° in patellar tilt yielded the greatest clinical benefit. These results support an individualized correction‐based strategy based on preoperative patellar tilt.

**Level of Evidence:**

Level III, retrospective comparative study.

## Graphical abstract

In 182 patients undergoing isolated MPFL reconstruction, the magnitude of patellar tilt correction—not a fixed postoperative threshold (〈20°)—was significantly associated with functional outcome (IKDC, Kujala; *p* < 0.001), supporting an individualized, correction‐based surgical strategy.

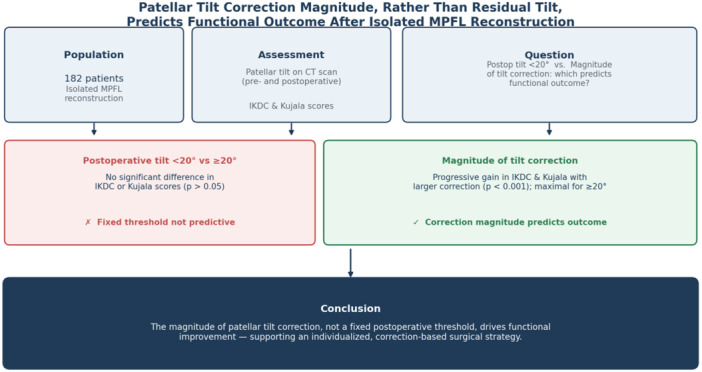

**Abbreviations**
BMI: body mass index
CD index: Caton–Deschamps index
IKDC: International Knee Documentation Committee score
LFU: last follow‐up
MPFL: medial patellofemoral ligament
QC: quadriceps contracted
QR: quadriceps relaxed
ROM: range of motion
TT–GT: tibial tubercle–trochlear groove distance

## INTRODUCTION

Patellofemoral instability is a common condition, particularly affecting young and active patients and is often associated with recurrent dislocations and functional impairment [15]. Its aetiology is multifactorial [37], involving both bony abnormalities and soft tissue insufficiencies, among which medial patellofemoral ligament (MPFL) deficiency plays a central role [1, 11, 15, 22, 23, 35, 36].

Isolated MPFL reconstruction has become a widely accepted surgical procedure for restoring medial patellar stability, with numerous studies demonstrating its effectiveness in reducing instability and improving functional outcomes [17, 33, 34]. Patellar tilt is recognized as a key radiological parameter in the assessment of patellofemoral instability [6, 7, 48]. A threshold of 20° on computed tomography (CT) scan measurements is commonly considered pathological, based on the work of Dejour et al. [8] and is frequently used to guide surgical decision‐making, as illustrated in the decision‐making algorithm of Fithian et al. [14].

However, the clinical relevance of this threshold remains controversial. While several studies have demonstrated that MPFL reconstruction reduces patellar tilt [2, 3, 19, 29, 38, 39, 49], few have investigated whether achieving a postoperative tilt below 20° is truly associated with improved functional outcomes. Moreover, it remains unclear whether the absolute postoperative value or the magnitude of correction is the most relevant determinant of clinical improvement.

The primary objective of this study was therefore to evaluate whether functional outcomes after isolated MPFL reconstruction are associated with achieving a postoperative patellar tilt below 20°. The secondary objective was to assess the relationship between the magnitude of tilt correction and functional improvement.

It was hypothesized that functional outcomes would be more strongly associated with the magnitude of correction than with achieving a predefined threshold value.

## MATERIALS AND METHODS

### Inclusion

This retrospective single‐centre study included all consecutive patients who underwent isolated MPFL reconstruction between September 2007 and July 2023. Inclusion criteria were skeletal maturity, a history of at least two patellar dislocations and persistent patellar instability for a minimum of 6 months. Exclusion criteria were trochlear dysplasia greater than Type A [8], patella alta (Caton–Deschamps index [CD index] > 1.2) [5], a tibial tuberosity–trochlear groove (TT–TG) distance >20 mm [9], previous knee surgery, a history of knee infection or advanced osteoarthritis.

The study was conducted in accordance with the Declaration of Helsinki and was approved by the institutional review board (IRB No. IRBN492015/CHUSTE).

#### Group constitution

Patients were classified according to postoperative patellar tilt, assessed with the quadriceps contracted (QC) and quadriceps relaxed (QR), into two groups (<20° and ≥20°). A threshold of 20° was used, as this value has been established as the pathological cutoff for patellar tilt by Dejour et al. [9]. Functional outcomes were then compared between the two groups.

### Surgical technique

**Figure 1:**
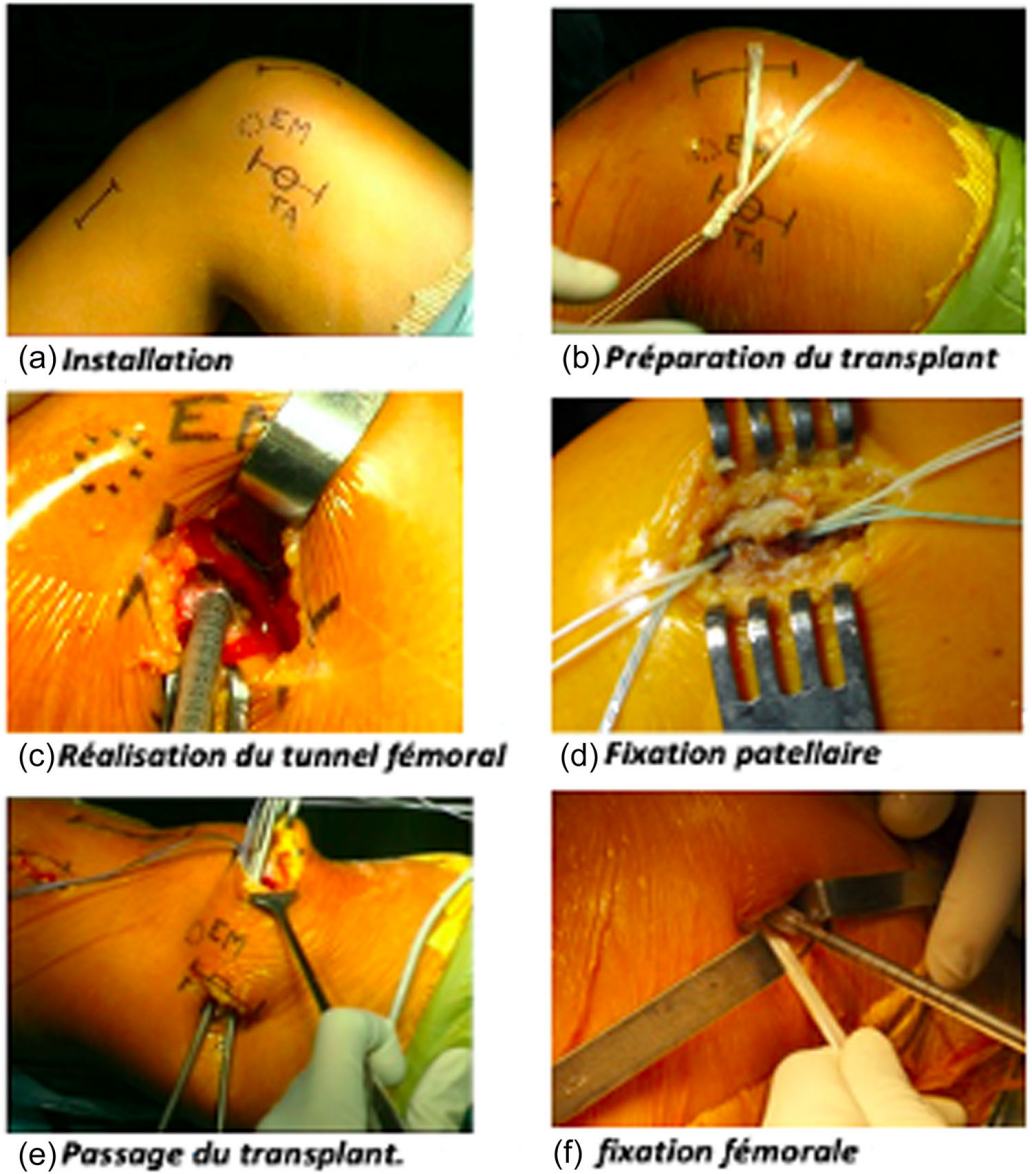
Surgical technique (Philippot et al. Study of patellar kinematics after reconstruction of the medial patellofemoral ligament.) (a) Installation. (b) Préparation du transplant. (c) Réalisation du tunnel fémoral. (d) Fixation patellaire. (e) Passage du transplant. (f) Fixation fémorale.

The surgical technique (Figure 1) is standardized among the various operators, responding to the different biomechanical studies conducted on the native MPFL [12, 13, 43, 44, 46]. The gracilis tendon is harvested to create the graft in accordance with the literature data [25, 47].

The graft is folded in half to create two equal limbs and sutured at the fold, resulting in a Y‐shaped configuration with a single femoral limb and two patellar limbs.

A medial femoral incision is made between the medial epicondyle and the adductor tubercle. The femoral insertion site is identified 10 mm distal to the adductor tubercle and 10 mm posterior to the medial femoral epicondyle, according to previous cadaveric studies [30, 37]. A 6‐mm diameter, 30‐mm deep blind femoral tunnel is then drilled over a guidewire under fluoroscopic guidance, confirming the criteria described by Schöttle et al. [40].

A longitudinal incision is made along the medial patellar border. Two 3.5‐mm bioabsorbable suture anchors (Twins Fix; Smith & Nephew) are inserted: one at the superior pole of the patella and the second at the junction of the upper and middle thirds.

A plane is developed between the superficial retinaculum and the joint capsule, allowing passage of the two patellar limbs, which are secured to the anchors. The femoral limb is then passed into the blind the blind tunnel. Graft tension was not controlled using a dynamometer; instead, tension was set based on clinical reduction alone to restore patellar alignment while avoiding overtensioning and excessive patellofemoral contact pressure. Final femoral fixation is achieved with an interference screw.

### Data collection

Preoperatively, demographic data, luxation number and functional scores were assessed at the initial consultation. Patients were reviewed postoperatively at 45 days, 3 months, 6 months and 1 year, and annually thereafter. Functional outcomes, including the International Knee Documentation Committee (IKDC) [18] and Kujala scores [24], were recorded at 6 months, 1 year and final follow‐up. A validated French adaptation of the Kujala score was used [20]; however, only a paediatric French version of the IKDC has been validated [27], with no adult French version available, which is acknowledged as a limitation.

Postoperative failure was defined as any recurrent patellar dislocation episode on the ipsilateral knee, regardless of whether revision surgery was performed.

### Radiographic assessment

The following parameters were measured on these radiographs:

- Patellar height via the CD index [5].
- Trochlear dysplasia according to D. Dejour's classification [8].
- Laurin angle [26].
- Merchant angle.

**Figure 2:**
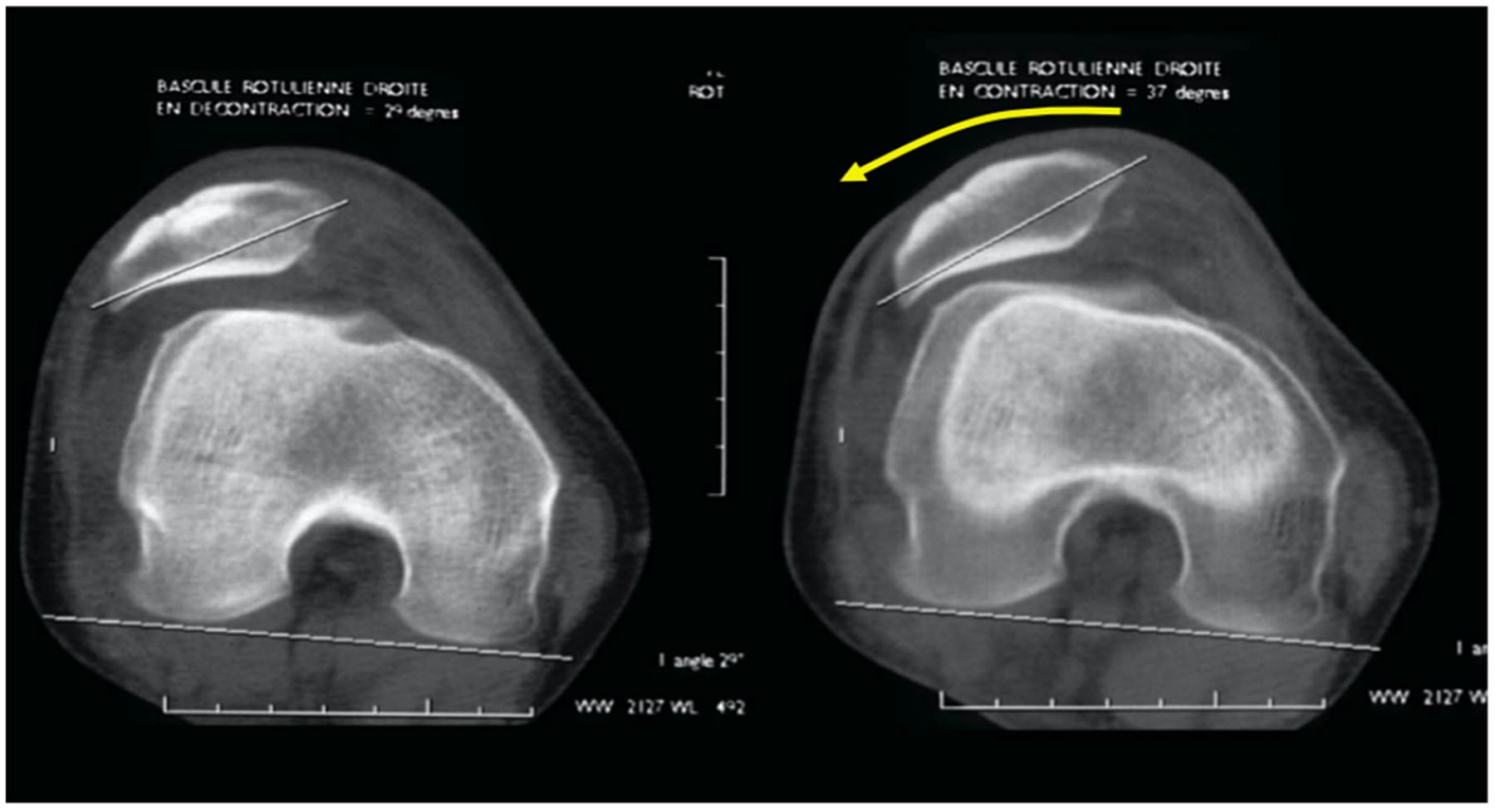
Patellar tilt measurement.

The initial CT‐based assessment and the one at 6 months postoperatively included:

- TT‐GT measurement**:** This measures the distance between the anterior tibial tuberosity and the bottom of the trochlear groove [9].
- Patellar tilt measurement (Figure 2)**:** This is assessed by superimposing two scan cuts, one passing through the long axis of the patella and the other through the reference trochlear cut (where the notch has a Roman arch shape). It forms the angle that the long axis of the patella makes with the posterior bicondylar plane [16, 41]. This CT‐based measurement of lateral patellar tilt has previously been shown to have excellent intra‐ and interobserver reliability (intraclass correlation coefficient >0.75) [4, 10], and all measurements in the present study were performed by a single trained observer following this standardized protocol.

### Statistical analysis

Statistical analyses were performed using SPSS software (SPSS Inc.). The significance threshold was set at *p* < 0.05. Continuous variables were expressed as mean ± standard deviation [min–max], and categorical variables as absolute and relative frequencies. The normality of distributions was assessed using the Shapiro–Wilk test and homogeneity of variances using Levene's test; parametric or non‐parametric tests were selected accordingly.

To address the primary outcome, patients were stratified into two groups based on their postoperative patellar tilt, measured under both QC and QR conditions: a group with a postoperative tilt below 20° and a group with a postoperative tilt of 20° or greater. Between‐group comparisons of continuous variables were performed using the unpaired Student's *t* test for normally distributed variables or the Mann–Whitney *U* test otherwise. Categorical variables were compared using the chi‐squared test or Fisher's exact test, as appropriate. The strength of association between functional outcomes (IKDC and Kujala scores) and both pre‐ and postoperative patellar tilt values under QC and QR conditions was assessed using Pearson or Spearman correlation coefficients, depending on data distribution.

To address the secondary outcome, patients were categorized according to the magnitude of postoperative tilt correction under QC and QR conditions (diffQC and diffQR), with subgroup analyses conducted in 5° increments. Differences in functional improvement across subgroups were compared using one‐way analysis of variance (ANOVA) followed by post hoc testing. This analysis was additionally applied to the overall cohort to examine whether the magnitude of tilt correction was associated with functional gains independently of preoperative tilt stratification.

Finally, a generalized linear model was constructed to evaluate whether the magnitude of tilt correction independently predicted functional outcomes (IKDC and Kujala scores at last follow‐up [LFU]) after adjustment for the following covariates: age, body mass index (BMI), preoperative IKDC and Kujala scores, pre‐ and postoperative patellar tilt under QC and QR conditions, pre‐ and postoperative tibial tuberosity–trochlear groove (TT–TG) distance and the CD index.

## RESULTS

**Figure 3:**
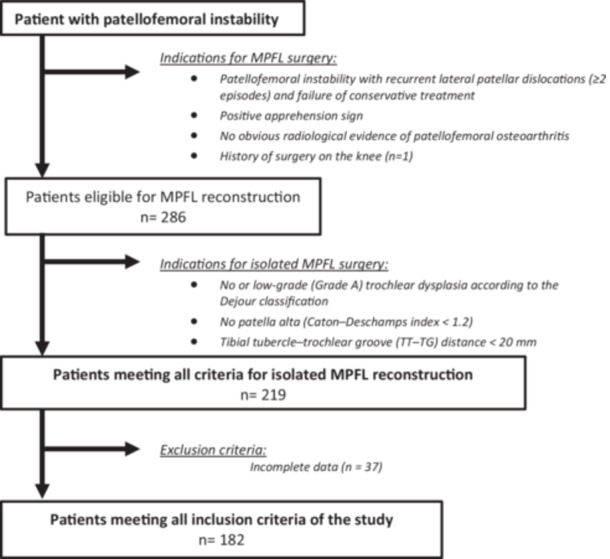
Flowchart. MPFL, medial patellofemoral ligament.

A total of 219 patients were enroled; 38 were excluded because of incomplete data and history of surgery on the operative knee (Figure 3, flowchart).

Finally, we analysed 182 patients: 103 women (56.6%) and 79 men (43.4%), with an average age at surgery of 23.58 years (± 10.27) and 3 ± 2 episodes of dislocations. The average follow‐up period was 6.4 years (± 3.9).

Demographic characteristics of the entire cohort and of each group are summarized in Table 1.

**Table 1:** Demographic characteristics of the total population.

Variable	Entire population *N* = 182	QC post < 20° *N* = 136 (74.7%)	QC post > 20° *N* = 46 (25.3%)	*p* value	QR post < 20° *N* = 164 (84.6%)	QR post > 20° *N* = 28 (15.4%)	*p* value
Age of surgery (year)	23.58 (± 10.27)	22.88 (± 8.72)	25.67 (± 13.77)	0.563	23.8 (± 10.65)	22.17 (± 7.25)	0.995
Gender							
Female	103 (56.6%)	58 (42.65%)	21 (45.65%)	0.854	66 (42.86%)	13 (46.43%)	0.886
Male	79 (43.4%)	78 (57.35%)	25 (54.35%)		88 (57.14%)	15 (53.57%)	
Laterality							
Right	91 (50%)	66 (48.53%)	25 (54.35%)	0.609	78 (50.65%)	13 (46.43%)	0.837
Left	91 (50%)	70 (51.47%)	21 (45.65%)		76 (49.35%)	15 (53.57%)	
BMI	23.63 (± 4.95)	23.54 (± 4.96)	23.93 (± 5.01)	0.835	23.44 (± 5.05)	24.76 (± 4.34)	0.115
Luxation number	3 (± 2)	3 (± 2)	3 (± 2)	0.29	3 (± 2)	3 (± 2)	0.77
IKDC preop	45.12 (± 13.62)	45.01 (± 13.22)	45.45 (± 14.91)	0.952	45.60 (± 13.46)	42.53 (± 14.45)	0.271
Kujala preop	55.48 (± 11.62)	54.76 (± 10.64)	57.63 (± 14.05)	0.383	55.55 (± 11.44)	55.11 (± 12.8)	0.658
Trochlea Dejour Type A	42 (28%)	30 (28.3%)	12 (30.0%)	0.743	35 (28.69%)	7 (29.17%)	0.863
CDindexpre	1.12 (± 0.14)	1.12 (± 0.134)	1.1 (± 0.159)	0.341	1.12 (± 0.142)	1.08 (± 0.13)	0.214
TT‐GTpre	15.93 (± 3.78)	15.93 (± 3.21)	15.96 (± 5.32)	0.679	15.79 (± 3.2	16.68 (± 6.08)	0.58

*Note*: Values are expressed as mean ± standard deviation or number (percentage).

Abbreviations: BMI, body mass index; CD index, Caton–Deschamps index; IKDC, International Knee Documentation Committee score; QC, quadriceps contracted; QR, quadriceps relaxed; TT‐GT, tibial tubercle–trochlear groove distance.

Forty‐six patients (25.3%) had a postoperative patellar tilt of 20° or greater under quadriceps‐contracted conditions and 28 (15.4%) under quadriceps‐relaxed conditions.

Patellar tilt was significantly reduced in both contracted (*p* < 0.001) and relaxed quadriceps (*p* < 0.001) between preoperative and postoperative assessments across all studied groups. The IKDC and Kujala scores were also significantly improved from preoperative to the LFU (*p* < 0.001). The results are presented in Tables 2 and 3.

**Table 2:** Comparative analysis of postoperative patellar tilt QC.

	**QC post < 20°**			QC post > 20°		
	Preoperative	Postoperative	*p* value	Preoperative	Postoperative	*p* value
Clinical scores						
IKDC	43.68 (± 19.83)	77.01 (± 20.98)	*p* < 0.001	44.83 (± 19.50)	73.56 (± 19.25)	*p* < 0.001
KUJALA	53.00 (± 14.00)	87.50 (± 11.00)	*p* < 0.001	52.00 (± 20.00)	87.00 (± 13.25)	*p* < 0.001
Patellar tilt						
QC	28.00 (± 9.25)	17.00 (± 5.00)	*p* < 0.001	34.00 (± 15.75)	22.00 (± 6.00)	*p* < 0.001
Quadriceps relaxed	24.00 (± 9.00)	15.00 (± 5.00)	*p* < 0.001	30.00 (± 15.50)	20.00 (± 5.75)	*p* < 0.001

*Note*: Values are expressed as mean ± standard deviation.

Abbreviations: IKDC, International Knee Documentation Committee score; QC, quadriceps contracted.

**Table 3:** Comparative analysis of postoperative patellar tilt QR.

	QR post < 20°			QR post > 20°		
	Preoperative	Postoperative	*p* value	Preoperative	Postoperative	*p* value
Clinical scores						
IKDC	43.68 (± 19.54)	75.86 (± 20.98)	*p* < 0.001	41.95 (± 24.43)	78.16 (± 25.86)	*p* < 0.001
KUJALA	53.00 (± 15.25)	87.00 (± 11.00)	*p *< 0.001	51.00 (± 14.50)	90.50 (± 13.25)	*p* < 0.001
Patellar tilt						
Quadriceps contracted	28.00 (± 10.50)	18.00 (± 4.00).	*p* < 0.001	43.00 (± 16.25)	25.00 (± 4.50)	*p* < 0.001
Quadriceps relaxed	25.00 (± 9.25)	16.00 (± 5.00)	*p* < 0.001	36.50 (± 13.00)	22.50 (± 6.00)	*p* < 0.001

*Note*: Values are expressed as mean ± standard deviation.

Abbreviations: IKDC, International Knee Documentation Committee score; QR, quadriceps relaxed.

The failure rate was 1.65%, defined as three new episodes of patellar dislocation. All three patients required revision surgery for graft rupture: two occurred following a road traffic accident and one during rugby practice.

1. Comparison between patients with a postoperative patellar tilt > and < 20°

The QC group included 136 patients presenting a tilt less than 20° (74.7%), while the QR group had 164 patients (84.6%). QC generates a lateral force vector on the patella, resulting in greater patellar tilt compared to the relaxed state, which accounts for the higher proportion of patients exceeding the 20° threshold under quadriceps‐contracted conditions.

All groups were comparable regarding demographic and preoperative clinical characteristics, except for the studied variables.

A comparative analysis was conducted between these two groups for the QC. The analysis was repeated for the relaxed quadriceps. The results are presented in Table 4.

**Table 4:** Comparative analysis of postoperative patellar tilt QC and QR.

Variable	QR post < 20° *N* = 164 (84.6%)	QR post > 20° *N* = 28 (15.4%)	*p* value	QC post < 20° *N* = 136 (74.7%)	QC post > 20° *N* = 46 (25.3%)	*p* value
IKDC LFU	73.91 (± 15.30)	72.10 (± 20.48)	0.715	74.20 (± 15.79)	72.46 (± 17.27)	0.58
Kujala LFU	86.05 (± 10.52)	87.32 (± 13.06)	0.191	85.88 (± 11.45)	87.30 (± 9.22)	0.721
Rom LFU	137.69 (± 10.44)	137.40 (± 9.91)	0.421	137.98 (± 10.62)	136.63 (± 9.49)	0.081
Failure						
Oui	3 (2.1%)	0 (0.0%)	>0.999	3 (2.3%)	0 (0.0%)	0.571
Non	146 (97.9%)	27 (100.0%)		128 (97.7%)	45 (100.0%)	
CD LFU	1.10 (± 0.13)	1.09 (± 0.14)	0.766	1.10 (± 0.13)	1.09 (± 0.16)	0.922
QCpre (°)	29.04 (± 7.81)	40.21 (± 10.43)	<0.001	28.67 (± 7.87)	36.93 (± 10.01)	<0.001
QRpre (°)	25.92 (± 7.23)	36.43 (± 8.17)	<0.001	25.93 (± 7.38)	32.26 (± 9.07)	<0.001
TT‐GTpost	15.49 (± 3.66)	15.95 (± 2.95)	0.494	15.31 (± 3.52)	16.33 (± 3.61)	0.185

*Note*: Values are expressed as mean ± standard deviation or number (percentage).

Abbreviations: CD index, Caton–Deschamps index; IKDC, International Knee Documentation Committee score; LFU, last follow‐up; QC, quadriceps contracted; QR, quadriceps relaxed; ROM, range of motion; TT‐GT, tibial tubercle–trochlear groove distance.

Between the two groups, there was no significant difference in functional scores at the LFU between a tilt greater than and less than 20° for QC (IKDC *p* = 0.58 and Kujala *p* = 0.721). The same results were found for QR (IKDC *p* = 0.715 and Kujala *p* = 0.191).

The preoperative patellar tilt for QC (28.67 [± 7.87] vs. 36.93 [± 10.01] *p* < 0.001) and QR (25.92 [± 7.23] vs. 36.43 [± 8.17] *p* < 0.001) was significantly different between the two groups.

2. Optimal patellar tilt correction

**Figure 4:**
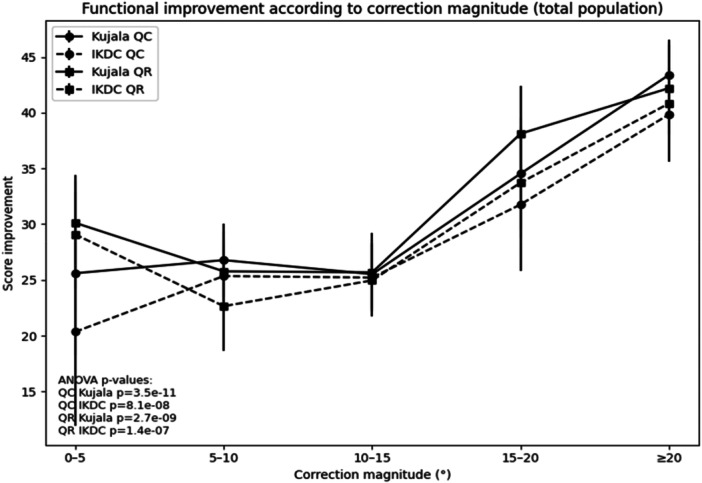
Functional improvement according to correction magnitude. ANOVA, analysis of variance; IKDC, International Knee Documentation Committee score; QC, quadriceps contracted; QR, quadriceps relaxed.

For the secondary analysis, patients were then divided into subgroups categorized by 5° increments of correction magnitude. Baseline characteristics were comparable between groups regarding demographic parameters (age, BMI, TT‐GT and CD index), whereas preoperative patellar tilt and functional scores significantly differed across groups, reflecting increasing baseline severity with higher correction magnitudes (Table 5 and Figure 4).

**Table 5:** Group characteristics according to the difference in tilt correction.

Group	*N*	IKDC pre	IKDC post	Kujala pre	Kujala post	QC pre (°)	QR pre (°)
Characteristics according to diffQC							
0–5	20	48.4 [27.6–78.2]	68.8 [31.0–99.0]	56.6 [43.0–100.0]	82.2 [45.0–100.0]	22.4 [13.0–45.0]	21.6 [12.0–36.0]
5–10	49	46.0 [20.7–80.5]	71.3 [26.0–94.0]	56.5 [37.0–83.0]	83.3 [42.0–99.0]	25.4 [14.0–34.0]	23.1 [10.0–37.0]
10–15	53	48.0 [18.4–73.6]	73.3 [21.0–95.0]	59.5 [36.0–86.0]	85.1 [37.0–100.0]	29.9 [19.0–46.0]	26.4 [17.0–46.0]
15–20	22	41.5 [19.5–80.5]	73.3 [21.0–94.0]	54.4 [40.0–82.0]	88.9 [69.0–99.0]	32.3 [21.0–42.0]	29.2 [18.0–38.0]
≥20	38	40.0 [25.3–57.5]	79.9 [41.0–99.0]	48.6 [40.0–79.0]	92.0 [80.0–100.0]	42.8 [28.0–60.0]	37.2 [13.0–52.0]
*p* values ANOVA		0.029	0.070	<0.005	<0.001	<0.001	<0.001
Characteristics according to diffQR							
0–5	25	43.2 [19.5–64.4]	72.2 [21.0–99.0]	54.3 [40.0–82.0]	84.4 [51.0–100.0]	26.2 [13.0–45.0]	20.9 [10.0–36.0]
5–10	48	47.5 [20.7–80.5]	70.1 [26.0–94.0]	58.5 [37.0–100.0]	84.3 [42.0–100.0]	26.8 [19.0–43.0]	24.2 [18.0–37.0]
10–15	51	50.5 [31.0–75.9]	75.4 [41.0–95.0]	60.4 [40.0–86.0]	86.1 [56.0–100.0]	27.8 [15.0–43.0]	25.7 [13.0–45.0]
15–20	31	37.8 [18.4–54.0]	71.7 [21.0–91.0]	48.8 [36.0–67.0]	86.9 [37.0–100.0]	35.1 [18.0–51.0]	31.6 [18.0–46.0]
≥20	27	41.4 [25.3–57.5]	82.3 [41.0–99.0]	50.1 [40.0–79.0]	92.3 [80.0–100.0]	43.6 [27.0–60.0]	39.6 [27.0–52.0]
*p* values ANOVA		<0.001	0.018	<0.001	0.021	<0.001	<0.001

*Note*: Values are expressed as mean [min–max].

Abbreviations: ANOVA, analysis of variance; CD index, Caton–Deschamps index; IKDC, International Knee Documentation Committee score; TT‐GT, tibial tubercle–trochlear groove distance.

The average difference in tilt pre‐ and postoperatively was 13.03 ± 7.92° for QC and 11.82 ± 7.37° for QR.

There was no correlation between the improvement in functional scores and the initial value of the tilt for QC (IKDC *p* = 0.956 and Kujala *p* = 0.875) and QR (IKDC *p* = 0.318 and Kujala *p* = 0.110). There was also no association between the functional scores improvement and the value of postoperative tilt for QC (IKDC *p* = 0.729 and Kujala *p* = 0.645) and QR (IKDC *p* = 0.725 and Kujala *p* = 0.904).

In the overall population, a progressive increase in functional improvement was observed with increasing correction magnitude (diffQC and diffQR). Both IKDC and Kujala scores demonstrated greater improvements with higher degrees of correction, with a marked increase beyond 15° and maximal gains observed for corrections ≥20°. This trend was consistent across both QC and QR analyses, as shown in Figure 4. Analysis of variance confirmed statistically significant differences between correction groups for all comparisons (all *p* < 0.001).

After adjustment for potential confounders, the magnitude of patellar tilt correction remained independently associated with functional improvement at final follow‐up. For contracted quadriceps, each additional degree of correction was associated with a 0.74‐point increase in IKDC improvement and a 0.64‐point increase in Kujala improvement, both *p* < 0.001. For relaxed quadriceps, each additional degree of correction was associated with a 0.62‐point increase in IKDC improvement and a 0.53‐point increase in Kujala improvement, both *p* < 0.001. These findings confirm that the degree of correction, rather than the absolute postoperative tilt value, was independently associated with better functional recovery after isolated MPFL reconstruction.

## DISCUSSION

The main finding of the present study was that functional improvement following isolated MPFL reconstruction was more strongly associated with the extent of patellar tilt correction than with the achievement of a specific postoperative patellar tilt threshold.

Although patellar tilt was significantly reduced postoperatively, bringing the tilt below the commonly accepted 20° threshold did not result in superior functional outcomes. This finding is consistent with previous studies, notably Sappey‐Marinier et al. [38], which questioned the relevance of this threshold as a determinant of clinical success.

In contrast, the results demonstrate that the magnitude of correction appeared more strongly associated with functional improvement than postoperative tilt values, with greater improvements observed in patients achieving larger differentials.

From a biomechanical perspective, these findings support the concept that the MPFL acts primarily as a passive mechanical restraint against lateral patellar translation rather than as a structure restoring normal patellofemoral kinematics [42]. As described by Amis et al. [1] and Desio et al. [11], the MPFL contributes significantly to medial stabilization in early flexion, functioning as a security belt against lateral displacement.

Therefore, the goal of MPFL reconstruction should not be to restore a ‘normal’ patellar alignment, but rather to prevent pathologic lateral translation and the sensation of subluxation. This point is essential: patellofemoral instability is a multifactorial condition [36, 37, 38, 39, 40, 41], influenced by limb alignment, patellar height, trochlear and quadriceps dysplasia and soft‑tissue imbalance. MPFL reconstruction therefore addresses only one element of the problem, acting primarily as a medial restraint.

Attempting to overcorrect patellar tilt in order to normalize imaging parameters may therefore be inappropriate and potentially harmful. Several biomechanical studies [28, 45], including those of Ostermeier et al. [32], have demonstrated that excessive tensioning or overcorrection during MPFL reconstruction can lead to increased medial patellofemoral contact pressures, potentially resulting in pain, stiffness and early medial compartment overload.

Clinically, these findings support a more conservative and individualized approach to correction, in line with the results reported by Klasan et al. [21]. Rather than targeting a fixed threshold or aiming for complete normalization, a patient‐specific correction strategy may be preferable, in agreement with the results reported by Ntagiopoulos et al. [31]. Surgeons should focus on achieving sufficient correction to restore stability. Finally, the greater improvements observed in patients with larger corrections may also reflect a greater potential for improvement in patients with more severe preoperative abnormalities, which should be considered when interpreting these results.

However, this study has several limitations that should be acknowledged. First, its retrospective single‐centre design introduces inherent selection bias. Second, graft tension was set based on clinical reduction alone rather than dynamometer control, which may introduce inter‐operator variability in tensioning and limit the reproducibility of the surgical technique. Third, QC during CT scanning was not objectively standardized, and variability in muscle activation between patients may have influenced tilt measurements. Finally, functional outcomes were assessed using patient‐reported outcome measures (IKDC and Kujala scores), which, although widely validated, remain subjective and may not fully capture objective functional recovery.

These limitations notwithstanding, this study provides meaningful insights into the relationship between patellar tilt correction and functional outcomes following isolated MPFL reconstruction, based on a relatively large cohort with a standardized surgical technique and systematic radiological assessment.

## CONCLUSION

The principal finding of this study is that functional improvement after isolated MPFL reconstruction is more strongly associated with the magnitude of patellar tilt correction than with attainment of a specific postoperative tilt threshold. Corrections exceeding 15° yielded the greatest clinical benefit. These results support an individualized correction‐based strategy based on preoperative patellar tilt, which may optimize outcomes.

## AUTHOR CONTRIBUTIONS

All authors contributed to the conception and design of the study. Data collection and analysis were performed by Ella George and co‐authors. The first draft of the manuscript was written by Ella George. All authors critically revised the manuscript, approved the final version and agree to be accountable for all aspects of the work.

## FUNDING INFORMATION

The authors have no funding to report.

## CONFLICT OF INTEREST STATEMENT

The authors declare no conflicts of interest.

## ETHICS STATEMENT

This study was conducted in accordance with the Declaration of Helsinki and was approved by the institutional review board of the University Hospital of Saint‐Étienne, France (IRB IRBN492015/CHUSTE).

## Data Availability

The data that support the findings of this study are available from the corresponding author.
